# Feasibility and cost-effectiveness of a multidisciplinary home-telehealth intervention programme to reduce falls among elderly discharged from hospital: study protocol for a randomized controlled trial

**DOI:** 10.1186/s12877-016-0378-z

**Published:** 2016-12-07

**Authors:** Alessandro Giordano, Gian Pietro Bonometti, Fabio Vanoglio, Mara Paneroni, Palmira Bernocchi, Laura Comini, Amerigo Giordano

**Affiliations:** 1 Operative Unit for Recovery and Functional Rehabilitation, Salvatore Maugeri Foundation IRCCS, Institute of Lumezzane (Brescia), Via Giuseppe Mazzini, 129, 25065 Lumezzane (Brescia), Italy; 2 Neurological Rehabilitation, Salvatore Maugeri Foundation IRCCS, Institute of Lumezzane (Brescia), Brescia, Italy; 3 Cardiac Rehabilitation Division, Salvatore Maugeri Foundation IRCCS, Institute of Lumezzane (Brescia), Brescia, Italy; 4 Telemedicine Service, Salvatore Maugeri Foundation IRCCS, Institute of Lumezzane (Brescia), Brescia, Italy; 5 Health Directorate, Salvatore Maugeri Foundation IRCCS, Institute of Lumezzane (Brescia), Brescia, Italy

**Keywords:** Fall, Prevention, Information Communication Technologies, Trial, Cost-effectiveness

## Abstract

**Background:**

Fall incidents are the third cause of chronic disablement in elderly according to the World Health Organization (WHO). Recent meta-analyses shows that a multifactorial falls risk assessment and management programmes are effective in all older population studied. However, the application of these programmes may not be the same in all National health care setting and, consequently, needs to be evaluated by cost-effectiveness studies before to plan this intervention in regular care. In Italy structured collaboration between hospital staff and primary care is generally lacking and the role of Information and Communication Technologies (ICT) in a fall prevention programme at home has never been explored.

**Methods and design:**

This will be a two-group randomised controlled trial aiming to evaluate the effects of a home-based intervention programme delivered by a multidisciplinary health team. The home tele-management programme, previously adopted in our Institute for chronic patients, will be proposed to elderly people affected by chronic diseases at high risk of falling at hospital discharge. The programme will involve the hospital staff and will be managed thanks to the collaboration between hospital and primary care setting. Patients will be followed for 6 months after hospital discharge. A nurse-tutor telephone support and tele-exercise will characterize the intervention programme. People in the control group will receive usual care. The main outcome measure of the study will be the percentage of patients sustaining a fall during the 6-months follow-up period. An economic evaluation will be performed from a societal perspective and will involve calculating cost-effectiveness and cost utility ratios.

**Discussion:**

To date, no adequately powered studies have investigated the effect of the Information and Communication Technologies (ICT) in a home fall prevention program. We aim the program will be feasible in terms of intensity and characteristics, but particularly in terms of patient and provider compliance. The results of the economic evaluation could provide information about the cost-effectiveness of the intervention and the effects on quality of life. In case of shown effectiveness and cost effectiveness, the program could be implemented into health services settings.

**Trial registration:**

ClinicalTrials.gov (NCT02487589)

## Background

About one-third of people over the age of 65 fall at least once a year [1]. Moreover people who have fallen are at higher risk of falling again [2] and show an increase in morbidity, mortality and healthcare utilization [2], which implies an increase in healthcare costs [3–5].

These facts emphasize the need to adopt measures to prevent falling in older persons.

Several fall prevention strategies including educational support, physical exercise and modification of environmental factors have been evaluated in studies [1] conducted in elderly people living in community.

In general, evidence suggests that interventions individually tailored to target risk factors seem to be more effective than those applied as a standard package [6].

Nevertheless, a systematic Cochrane reviewed 159 randomized trials involving 79,193 older persons living in community, failed to establish which fall prevention interventions are more effective, but confirmed the positive effect of the intervention in persons with history of falling or in those at higher risk [7].

However, it should be emphasized that even when programs for fall prevention have been successful in a controlled research setting, the transfer of similar protocols to real world settings has not always resulted in fall prevention [8]. Both patient and provider compliance with the protocol, as well as expertise in delivering services, such balance training, are felt to be possible barriers for a successful implementation [9].

In this scenario the role of Information and Communication Technologies (ICT) in a home fall prevention program could be crucial. Telephone support, telemonitoring and tele-exercise program could improve patient’s compliance, as well as lead to a personalized management of the patient’s risk profile allowing the identification of a well-defined model, e.g. a home fall prevention program.

The role of ICT in a home fall prevention program has never been explored and besides this, studies focusing on economic evaluations in the context of a multidisciplinary intervention program are lacking [10–14].

This article describes the design of a randomized controlled trial aiming at evaluating the efficacy of a multidisciplinary intervention program, based on a tele-health home treatment, in elderly patients with high risk of falling discharged from a rehabilitation medical setting and living in community.

## Methods

### Study design and randomization

**Fig. 1:**
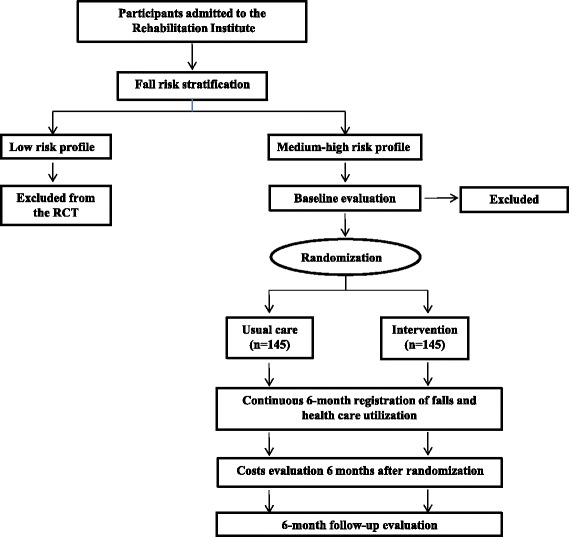
Design of the study

This study is a randomized controlled trial (RCT) with 6-month prospective follow-up evaluating the effect of a fall prevention program in a population with high risk of falling discharged home after rehabilitation. The Technical and Scientific Committee (CTS 04/2012) and Ethics Committee of the IRCCS Salvatore Maugeri of Pavia has approved the study design (CE 973 02/2014) and protocol; informed consent will be signed by patients at time of the hospital discharge. Figure 1 shows the design of the study.

Each amendment to the protocol will be discussed with the Ethics Committee.

The fall risk profile will be assessed in all participants 7–10 days before the hospital discharge. Medium/high fall risk profile will be defined by a history of fall within the last 12 months and/or by a Berg Balance scale score ≤ 45, and/or patients with at least one fall event during in-hospital stay. Patients with a low-risk of recurrent of falling will be excluded from the RCT. After having signed the informed consent, participants in medium/high-risk group will be allocated into either the control or intervention group (1:1 allocation ratio) using a computer-generated random allocation sequence concealed from researches (http://www.randomization.com). Staff not involved in the study will undertake the sequence and concealment. People allocated to the control group will receive usual care, while people in the intervention group participate to the 6-month multifactorial falls prevention program. During the follow-up period the incidence of falls and volumes of health care utilization will be recorded in both groups.

Health staff will be unblinded to participant enrollment. All data will be managed and stored by an independent call center.

### Study population

Participants will be patients of both sex, aged 65 years or over, living in community, admitted to the Rehabilitation Institute of Salvatore Maugeri Foundation, IRCCS Lumezzane (Brescia) for a usual period of rehabilitation.

Only patients with a medium-high risk profile of recurrent falling discharged home after rehabilitation will be considered eligible. Exclusion criteria will be: inability to sign the informed consent, presence of cognitive impairment Mini-Mental State Examination (MMSE) score (less than 18 or < 24 in patients living without a caregiver), living in a nursing home, permanently bedridden, or fully dependent on a wheelchair. We will exclude also patients affected by cancer, neurological impairment, including perceptual (neglect) and language limitations (aphasia).

### Intervention

The intervention will consist of a multifactorial falls prevention program implemented by a physiotherapist and will include:

1. **an individual home exercise program** with strength, balance and walking components based on the Otago Exercise Program [15, 16] widely used in more randomized clinical trials as a valid instrument to reduce fall incidence. The mainstay of Otago protocol is focused on implementing legs strength muscles mainly involved in gait and balance. The strengthening exercise are focused on major lower limb muscles: knee flexors, knee exstensors and hip abductors, which are particularly important for functional movements and walking, and ankle dorsiflexor and plantarflexor muscles involved in recovering balance. Due to the high percentage of frail patients admitted in our wards, we will need to adapt this protocol to our population and therefore we will divide patients in two different intervention subgroups (high and low intensity group) including the same exercises with different workload (Tables 1 and 2).

**Table 1:** Strengthening exercises

	Both groups	
1. Knee extensor (front knee strength)	Ankle cuff weights are used to provide resistance to the muscles and 10 repetitions of each exercise are carried out	
2. Knee flexor (back knee strength); Hip abductor (side hip strength)		
	Low intensity group	High intensity group
3. Ankle plantar-flexors (calf raises)	10 repetitions, hold support	10 repetitions, no support
4. Ankle dorsi-flexor (toe raises)

**Table 2:** Balance retraining exercises

	Low intensity group	High intensity group
Knee bends	10 repetitions, hold support	10 repetitions, hold support
Backwards walking		10 steps, 4 times, hold support
Walking and turning around		Walk and turn around twice, use walking aid
Sideways walking		10 steps, 4 times use walking aid
Tandem stance (heel toe stand)	10 seconds, hold support	10 seconds, no support
Tandem walk (heel to walk)		10 steps, hold support, repeat 10 times
One leg stand		10 seconds, hold support
Hell walking		10 steps, 4 times, hold support
Toe walk		10 steps, 4 times, hold support
Sit to stand	5 stands, 2 hands for support	5 stands, one hand or 10 stands, 2 hands for support
Stair walking	As instructed	As instructed
  Group selection will be determined by the research physiotherapist and administered after a baseline assessment. As in Otago, we will use Four Test Balance Scale as screening test to define the group selection. People able to complete the test as recommended (i.e. without some aid by operators, completing the four steps proposed characterized by growing difficulties in balance) will be included in the high intensity group. People performing less than three steps will be assigned to the low intensity group (Fig. 1).
  According to Otago program, we will propone exercises focused on improving balance and muscle strength, recommending the patient to have regular walk at least two times a week for at least 30 min. Participants will be advised to undertake their exercise program at least three times a week. Before leaving the hospital participants will receive a booklet with instructions for each exercise prescribed and ankle cuff weights to provide resistance for the strengthening exercises.
2. **health care assistance**
  A nurse tutor (NT) will follow up patients enrolled in the study with periodic phone called planned at least once a week. NT will promote health education support on fall prevention for patient and family, check out drug therapy adherence, collect new symptoms and concerns about current pharmacological. All personal and clinical patient’s data will be recorded on a web platform accessible in real time (Teleriab) to physicians, NT or physical therapists participating in the study.
  To help people to adhere to the program a research physiotherapist will follow the patients with training sessions through videoconference once a week during the first 3 months of the program, twice a month during the leading 2 months and monthly during the last month period. The physiotherapist will monitor the exercise session in real time tailoring the treatment to each single patient to avoid loss of adherence, and improving injury risk minimization strategies for patients at high risk.
  A software Platform of telemedicine (Teleriab), designed to offer telerehabilitation services, will be used to allow communication between patients and medical or paramedical team. Through this web platform the health staff will realize phone calls and video contacts with one or more patients contemporary (with a maximum of eight videoconferences simultaneously) or consecutively respecting the privacy of the patients. People owing a computer with an internet connection at home will receive an entry password to Teleriab once randomized to the group of intervention. People without a personal computer who live in a geographic area compatible with Internet Key connection will receive a netbook with a pre-installed “client” Teleriab version and an USB internet key. Teleriab leads an automatically access to internet once preinstalled. For patients without a their-own computer and without internet connection, a netbook with a preinstalled client version of Teleriab and an activated ADSL contract service will be provided until the end of the study.

### Control group

Although in Italy the guidelines have been released in 2007 [17], a systematic multifactorial fall risk prevention has not yet been implemented by general practitioners (GPs) or hospitals. Usual practice after a fall consists mainly of treatment of the consequence of a fall. Indeed, hospital physicians, specialists or general practitioners do not systematically address the patient’s risk behavior.

Participants in the control group will receive usual care by their GP. Before hospital discharge, we will provide control participants with written recommendation on fall risk factors. The same document will be sent to the GP. Once a month, participants in the control group will receive a phone call to check for incidence of falls, related complications and drug therapy.

## Measurements

### Baseline assessment

Fall risk stratification will be performed in all potentially eligible patients using a Berg Balance Scale [18]. We will consider at medium-high risk of falls patients with a Berg Balance scale score ≤ 45 and/or at least one episode of fall within the last 12 months, as well as people presenting at least one fall episode undertaken during hospitalization in our Institute. Cognitive state will be evaluated using Mini Mental Test Examination (MMSE) [19].

In patients who have signed the informed consent, fear of falling, gait and balance problem, functional status and quality of life is assessed. Fear of falling will be measured by the Italian version of Falls Efficacy Scale (FES) [20], gait and balance by Timed Up and Go Test (TUG) [21] or Balance Evaluation System’s Test (BEST) in patients with Parkinson disease, functional state by Activities Daily Living scale (ADL), Barthel Index [22], Instrumental Activity Daily Living scale (IADL) [23] and quality of life by the EQ-5D questionnaire. Daily doses of drugs considered correlated with fall risk such as antihypertensive drugs, diuretics, beta-blocker, calcium antagonists, nitrates, antiarrhytmics, insulin, oral hypoglycemic drugs, neuroleptics, antidepressives, antiepileptic and dopaminergic and thyroid drugs will be also recorded.

The same operators previously involved in baseline evaluations will collect them at the end of 6-month follow-up as well.

### Follow-up

Participants will be followed-up for 6 months after randomization. To ensure blinding during data collection, information and measurements will be collected by phone by an independent call center, whose operators have been trained to administer questionnaires and will be unaware of the group allocation. During this period patients will be monitored monthly about incidence of falls and related complications, clinical status, healthcare utilization, adherence to clinical recommendations and drug therapy modifications.

### Outcome measures

The primary outcome will be the proportion of fallers in the 6-month period after randomization. The secondary falls outcomes are the time between the first fall and the recurrent falls during follow-up and the percentage of patients sustaining two or more falls. A fall will be defined as “an event which results in a person coming to rest inadvertently on the ground or other lower level “ [24].

Other secondary outcomes include: changes in functional status (ADL, IADL and Barthel index), gait and balance measures (TUG or BEST test) and quality of life (EQ-5D).

The economic evaluations will be a combination of a cost-effectiveness and a cost-utility analysis [25] see list; we anticipated references [26]. The primary outcome measure for the cost-effectiveness analysis will be the percentage of people sustaining a fall during the 6-month follow-up. Within the cost-utility analysis the effects will be measured in terms of generic health-related quality of life descriptions, measured according to the standard Italian version of the EQ-5D [26], a self-administered questionnaire administered at baseline and after 6 months.

All healthcare and patient costs will be measured by means of a cost diary [27], in which participants continuously will record volumes of healthcare utilization during the 6 month follow-up period. In monthly telephone interviews, participants will be asked whether they had received care (or devices or services) or not. If participants will answer the question on a particular topic affirmatively, they will be asked to indicate the number of visits or details about the specific type of healthcare utilization. Data management will be organized “in-house”. Feasibility of the intervention program will be evaluated assessing participants compliance with the intervention protocol.

### Sample size

Sample size calculation has been based on the results of an observational study conducted in our Rehabilitation Institute in a sample of 179 patients. They were over 65 years old, medium-high fall risk profile, consecutively discharged from January to June 2013 and with a 6-month fall rate of 22.2%.

Consequently, we have estimated that 290 patients (145 in each group) are needed to detect a reduction of 40% of fallers in the intervention group with respect to the control one, with a power (1-beta) of 80%, alpha of 0.05 and an expected dropout rate of about 6%.

### Data analysis

Data will be primarily analyzed according to the intention-to-treat principle.

An on-treatment analysis will be subsequently performed to assess whether protocol deviations have caused bias. Participants with documented deviation from the study protocol (i.e. patients in the intervention group who did not receive the entire intervention or participants in both groups with incomplete follow-up data) will be excluded from this analysis. Chi-square analysis will be used to compare the proportion of fallers and non-fallers between groups. Cox proportional hazards regression will be conducted with time to first fall within 6 months of follow-up as outcome measure with age, sex, anthropometric data, prevalent disease, comorbidities, residual disability, marital status, social and economic status, MMSE and baseline variables as covariates. Multiple linear regression analyses will be used to compare differences in the other secondary outcomes at 6 months follow-up between groups. ANOVA with repeated measures will be used to analyze differences between groups from baseline to follow-up. Differences in baseline characteristics between groups will be examined using parametric (*T*-test) and non-parametric (Chi-square) tests. No ad interim analysis will be performed.

The economic evaluations will be conducted from a societal perspective and involve calculating cost-effectiveness ratio. The incremental costs and effects of the intervention will be compared with control group. The alfa level for all analyses is set at 0.05. Statistical analyses will be performed using STATA 13 program.

### Progress of the study

Recruitment of eligible subjects started in April 2014 and ended in December 2015, resulting in a total of 290 patients enrolled in the trial. The follow-up will end in June 2016 and then data-analysis will be initiated.

## Discussion

The main strengths of this study are the target population and the administration of the prevention program.

Despite evidence of the effectiveness of falls prevention activities for community-dwelling older people, the applicability of these interventions has not, to date, been specifically evaluated in elderly population affected by multiple comorbidity, discharged from a multispecialistic rehabilitation medical setting. The inclusion of patients with mild-moderate cognitive impairment represents a further originality. In fact, most studies conducted in community-dwelling patients have not specifically addressed older adults with cognitive impairment, and the effectiveness of fall prevention interventions in this population is not known [9]. Elderly adults with cognitive problems are one of the most vulnerable sectors of our society with clear mental, social, and physical disadvantages. They are more likely to experience falls, and experience further mobility decline due to having fallen; therefore it is urgent to identify evidence based interventions for reducing the risk of falls and related injuries in people with cognitive impairment.

Finally, an additional strength of the study is the administration of a fall prevention program supported by the new home-based technologies. This may contribute to a new approach to prevent and treat fall risk in this population. To date, no adequately powered studies have investigated the effect of the Information and Communication Technologies (ICT) in a home fall prevention program. We aim the program will be feasible in terms of intensity and characteristics, but particularly in terms of patient and provider compliance, which represent a possible barrier in successful implementation of fall prevention strategies [9].

Furthermore, the results of the economic evaluation could provide information about the cost-effectiveness of the intervention and the effects on quality of life. In case of shown effectiveness and cost effectiveness, the program could be implemented into health services settings.

All results from the study will be communicated by publication without any restriction.

### Limitation

The main potential critical aspect of our study is the sample size calculation. Epidemiological data on prevalence and fall rate incidence of subjects with a high fall risk profile coming from a multispecialistic rehabilitation setting are lacking. Consequently, we powered the study on the fall rate observed in a small number of patients discharged from our Institute that we hope will be confirmed in the usual care group.

## Abbreviations

BEST: Balance evaluation system’s test
FES: Falls efficacy scale
GPs: General practitioners
IADL: Instrumental activity daily living scale
ICT: Information and communication technologies
MMSE: Mini-mental state examination
NT: Nurse tutor
RCT: Randomized controlled trial
TUG: Timed up and go test
WHO: World Health Organization
